# Time-frequency signatures evoked by single-pulse deep brain stimulation to the subcallosal cingulate

**DOI:** 10.3389/fnhum.2022.939258

**Published:** 2022-08-18

**Authors:** Ezra E. Smith, Ki Sueng Choi, Ashan Veerakumar, Mosadoluwa Obatusin, Bryan Howell, Andrew H. Smith, Vineet Tiruvadi, Andrea L. Crowell, Patricio Riva-Posse, Sankaraleengam Alagapan, Christopher J. Rozell, Helen S. Mayberg, Allison C. Waters

**Affiliations:** ^1^Private Practice, Tucson, AZ, Pima; ^2^Departments of Psychiatry, Neuroscience, Neurology, Neurosurgery and Radiology, Nash Family Center for Advanced Circuit Therapeutics, Icahn School of Medicine at Mount Sinai, New York, NY, United States; ^3^Department of Psychiatry, Schulich School of Medicine and Dentistry, London, ON, Canada; ^4^Department of Biomedical Engineering, Duke University, Durham, NC, United States; ^5^Emory University School of Medicine, Atlanta, GA, United States; ^6^Department of Biomedical Engineering, Georgia Tech and Emory University, Atlanta, GA, United States; ^7^Department of Psychiatry and Behavioral Sciences, Emory University School of Medicine, Atlanta, GA, United States; ^8^School of Electrical and Computer Engineering, Georgia Institute of Technology, Atlanta, GA, United States

**Keywords:** deep brain stimulation, subcallosal cingulate, single pulse electrical stimulation, time frequency analyses, treatment resistant depression (TRD), inter-trial phase clustering, stimulation evoked potential, perturbation mapping

## Abstract

Precision targeting of specific white matter bundles that traverse the subcallosal cingulate (SCC) has been linked to efficacy of deep brain stimulation (DBS) for treatment resistant depression (TRD). Methods to confirm optimal target engagement in this heterogenous region are now critical to establish an objective treatment protocol. As yet unexamined are the time-frequency features of the SCC evoked potential (SCC-EP), including spectral power and phase-clustering. We examined these spectral features—evoked power and phase clustering—in a sample of TRD patients (*n* = 8) with implanted SCC stimulators. Electroencephalogram (EEG) was recorded during wakeful rest. Location of electrical stimulation in the SCC target region was the experimental manipulation. EEG was analyzed at the surface level with an average reference for a cluster of frontal sensors and at a time window identified by prior study (50–150 ms). Morlet wavelets generated indices of evoked power and inter-trial phase clustering. Enhanced phase clustering at theta frequency (4–7 Hz) was observed in every subject and was significantly correlated with SCC-EP magnitude, but only during left SCC stimulation. Stimulation to dorsal SCC evinced stronger phase clustering than ventral SCC. There was a weak correlation between phase clustering and white matter density. An increase in evoked delta power (2–4 Hz) was also coincident with SCC-EP, but was less consistent across participants. DBS evoked time-frequency features index mm-scale changes to the location of stimulation in the SCC target region and correlate with structural characteristics implicated in treatment optimization. Results also imply a shared generative mechanism (inter-trial phase clustering) between evoked potentials evinced by electrical stimulation and evoked potentials evinced by auditory/visual stimuli and behavioral tasks. Understanding how current injection impacts downstream cortical activity is essential to building new technologies that adapt treatment parameters to individual differences in neurophysiology.

## Introduction

### Background

There is growing scientific and clinical interest in the effect of single pulse electrical stimulation on the brain. This technique of perturbation mapping involves punctuated current injection to a circuit or cortical node using invasive (e.g., deep brain stimulation; DBS) or non-invasive methods (e.g., transcranial magnetic stimulation; TMS). Electrical perturbation of the living human brain elicits a temporal-spatial cascade of electrophysiological activity that appears sensitive to change in stimulation parameters, such as the precise location of stimulation in the brain. When this activity is averaged over repeated electrical pulses, a stereotyped series of spatial-temporal components are observed as an evoked potential. Importantly, DBS evoked potentials are coherent and reliable on the level of individuals (Waters et al., 2018), and are thus amenable to the development of patient-specific applications, such as confirmation of optimal surgical targeting. Precision targeting has been linked to the efficacy of subcallosal cingulate (SCC) DBS for treatment of depression (Riva-Posse et al., 2018). Understanding how the precise location of current injection impacts downstream cortical activity is essential to building new technologies that harness perturbation-based mapping approaches to confirm optimal therapeutic target engagement over the course of treatment.

A definitive biophysical explanation for evoked responses to single pulse stimulation is still unclear and may vary by scale (i.e., LFP, ECOG, EEG). Nevertheless, perturbation maps convey information that can be exploited to advance the clinical science of neuromodulation and to interrogate human brain networks (Fox et al., 2012; Entz et al., 2014; Sarasso et al., 2015; Borich et al., 2016; Solomon et al., 2018; Keller et al., 2018; Yu et al., 2019; Baker et al., 2002; Massimini et al., 2005). Stimulation-evoked brain responses are most frequently examined in the time domain (i.e., event related potentials, ERP) which ignores oscillatory features of neural activity like frequency, phase, and amplitude (Makeig et al., 2004). These spectral features are evident across spatial scales and species (Narayanan et al., 2013; Cohen, 2014; Robble et al., 2021), and a summation of spectral features—especially evoked power and phase consistency—contributes to manifest ERPs (Penny et al., 2002; Luu et al., 2004; Shah et al., 2004; Fuentemilla et al., 2006; Hanslmayr et al., 2007; Klimesch et al., 2007; Trujillo and Allen, 2007). Spectral metrics are also highly relevant to the study of depression pathophysiology because oscillation frequency and phase is critical to facilitating information multiplexing between and within brain networks. Spectral metrics also vary over time and examining dynamic frequency, phase, and amplitude is typically referred to as time-frequency analysis. This focus on examining brain activity in the time-frequency domain is also more compatible with the analytic techniques used with non-human animals, facilitating cross-species comparisons and interpretations (Cohen, 2011b; Basu et al., 2019). Amenability to cross-species comparisons is particularly relevant to understanding the effects of direct electrical stimulation to the brain since human trials are sparse and often costly. Altogether, applying time-frequency analysis to the investigation of stimulation-evoked responses can provide unique information that is obscured by conventional ERP analyses, facilitate cross-species comparisons and reveal biophysically plausible features relevant to functional brain networks.

### Present study

In an effort to expand upon prior work examining SCC stimulation evoked potentials, we focus our analyses on the ERP, evoked power, and inter-trial phase consistency (ITPC) as our primary neural measures. This study aimed to discover time-frequency signatures evoked by SCC stimulation in eight patients undergoing DBS for treatment resistant depression (TRD). We test the hypothesis that time-frequency features of perturbation map will vary as a function of DBS location across a dorsal-ventral axis of the SCC target region, which may reflect mechanisms of neuronal communication that are disrupted with precise targeting of white matter elements in the SCC region.

## Materials and methods

### Participants

Subjects (*n* = 8; four males) were patients in a study of SCC DBS safety and efficacy for treatment of TRD (clinicaltrials.gov #NCT01984710) who underwent DBS surgery between 2015 and 2019. Inclusion and exclusion criteria for the parent study were identical to Holtzheimer et al. (2012) and Riva-Posse et al. (2018) and summarized in Supplementary Materials. Briefly, participants suffered from severe major depressive disorder and had failed multiple treatments, including medication, psychotherapy, and electroconvulsive therapy. Subjects ranged in age from 28 to 70 (mean: 53.1, *SD*: 14.3). One subject was left handed. All participants provided written informed consent to participate in this research, which was approved by the Emory University Institutional Review Board and the US Food and Drug Administration under an Investigational Device Exemption (IDE # G130107 held by H.S.M) and was monitored by the Emory University Department of Psychiatry and Behavioral Sciences Data Monitoring Board. Additional sample characteristics, including depression severity scores (Hamilton, 1960) at baseline and at the time of study participation, are provided in Supplementary Material.

### Tractography guided implantation

**FIGURE 1:**
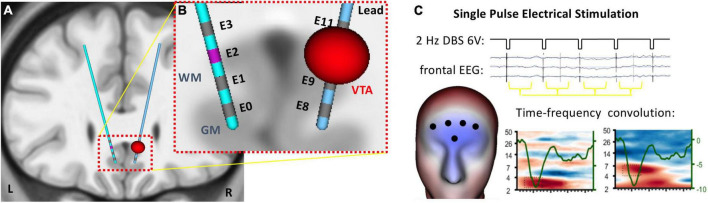
Single pulse electrical stimulation of the subcallosal cingulate (SCC) target for deep brain stimulation. **(A)** Four contacts span the SCC target region on bilateral DBS electrodes. **(B)** EEG was recorded on the head surface during single pulse electrical stimulation at each contact on the DBS leads. **(C)** Analytic window was coincident with the SCC-EP (∼100 ms) detected in frontal channels.

Procedures for tractography guided surgical targeting and post-operative verification follow Riva-Posse et al. (2018). An Activa PC + S™ pulse generator (Medtronic, Minneapolis, MNI) drove bilateral DBS leads (model 3387), each with 4 contacts (1.5 mm inter-contact spacing), which were implanted in the SCC region (Figure 1A) using a prospective connectomic approach and StimVision software (Noecker et al., 2017). This approach uses patient-specific deterministic tractography and anatomical images to optimize placement of the contact at the confluence point of four white matter fibers (Riva-Posse et al., 2014). In brief, magnetic resonance imaging data, (high-resolution T1 structural and diffusion-weighted) are acquired for each individual on a Siemens 3T Tim-Trio scanner (Siemens Medical Solution, Malvern, PA). Following surgery, high-resolution computed tomography (LightSpeed16, GE Medical System) images are used to verify that the contacts used for therapeutic stimulation respect to tractography.

### Experimental procedures

Patients were fitted with a 256-channel Hydrocel Geodesic Sensor Net (MagStim-EGI, Eugene, OR) and seated in a climate controlled room. A chin rest was used to reduce motion artifacts. Patients were instructed to relax and allow their mind to wander. A series of eight conditions, each 2.5–3 min of stimulation, involved simultaneous EEG recording and unilateral stimulation from different locations in the SCC target region (i.e., ventral, mid-ventral, mid-dorsal or dorsal contacts on each lead). All conditions used a monopolar configuration for stimulation of 6 V with a 90 μs pulse width at 2 Hz (Figure 1B). Conditions were not randomized. In conditions 1–4, stimulation was delivered to the left hemisphere from the ventral-most to dorsal-most contact, respectively. Conditions 5–8 followed the same pattern with stimulation delivered to the right hemisphere. Patients were informed as to the start and end of each condition but were blind to parameter settings. Stimulation parameter changes were made by a physician team member using the Medtronic clinical programmer. For individual patients, testing was conducted at different times in treatment. Four patients participated after 4 weeks of therapeutic stimulation and four patients participated after 6 months of stimulation (Supplementary Table 1). For one participant (Patient 2), experimental procedures were interrupted resulting in one condition recorded on a subsequent day.

### EEG preprocessing

Recordings were from a NetAmps 400 amplifier (MagStim-EGI, Eugene, OR) with an online reference near the vertex (1,000 Hz sampling rate). In four of the eight recordings, 1–3 bad electrodes (of 256) were identified manually and spherically interpolated; none were in the frontal montage used for statistical analysis. One subject (Patient 3) had high impedance in one of the implanted electrodes (also throughout the parent study). That contact was excluded from the experimental procedures for that subject, only. Electroencephalogram (EEG) were then re-referenced to the average of all electrodes. A 2–50 Hz bandpass zero-phase shift FIR filter was applied. Hampel outlier rejection in the frequency domain was the primary correction for stimulator artifacts specifically. The spectral outlier rejection by Hampel filtering has the advantage of preserving phase-relationships in the signal, and has demonstrable efficacy for reducing stimulation artifacts (Allen et al., 2010). Briefly, Hampel filtering involves rejecting spectral outliers using a sliding window. The user selects the frequency window width (*N* = 2) and outlier criterion for rejection (*t* = 5), then spectral bins identified as outliers are replaced with the average of their neighbors. Manual rejection of artifactual independent components analysis was used for other non-neurogenic artifacts (Smith et al., 2017), and any residual stimulator artifacts. Artifact is a substantial concern in these recordings, and aggressive multistage processing aligns with the recommendations of a recent discussion on the topic (Lio et al., 2018).

### EEG analyses

**FIGURE 2:**
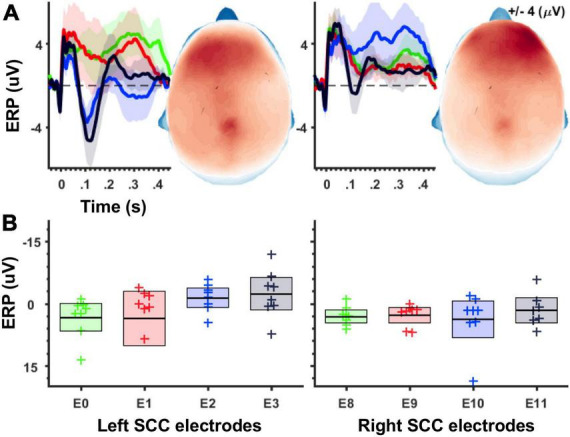
Magnitude of unilateral stimulation evoked potentials increases along the ventral-to-dorsal axis of the SCC target region. **(A)** Grand average waveforms for stimulation-evoked potentials. Shaded areas depict 95% confidence intervals after 1,000 bootstraps. Left panel shows ERPs following left SCC stimulation, and right panel shows ERPs following right SCC stimulation. Topographic plots show ERP magnitude integrated over 50–150 ms time window. **(B)** Boxplots depicting average ERP amplitudes (averaged across 50–150 ms, electrode montage shown in Figure 1C) separately for stimulation location. Left panel shows averages for left-SCC stimulation, right panel shows averages for right-SCC stimulation.

Average evoked-potentials. Epochs time-locked to the DBS pulse were cut and averaged to produce a mean time-series for each individual and in response to stimulation from each of eight contacts. A grand average is plotted for illustration in Figure 2.

Time-domain data were convolved with a family of Morlet wavelets to produce the time-frequency (TF) metrics of interest: ITPC and phase-locked TF power. The family of wavelets included 30 logarithmically spaced wavelets of varying frequency from 2 to 50 Hz, and with a varying number of cycles from 3 to 10 (higher frequencies with more cycles; e.g., Cohen, 2014). Time-frequency power was normalized (*Z*-score) relative to a -50 to -10 ms prestimulation baseline consistent with previous work (Waters et al., 2018).

### Tissue activation and white matter density

The DBS contact location was identified in native T1 space based on a high-resolution postoperative CT image that aligned to native T1 space using a linear registration toolbox (3dAllineate, AFNI: Analysis of Functional NeuroImages, Cox, 1996). The patient-specific volume of tissue activated (VTA) was then generated by electrical DBS field model on identified contact location for this study using the StimVision software toolbox with the following parameters: 130 Hz, 90 μs, and 6V (Noecker et al., 2018). The detailed methodology for DBS activation volume is described in Chaturvedi et al. (2013). Brain tissue segmentation was performed using a multichannel tissue classification algorithm (FAST, FMRIB)^1^ to calculate the probability of gray matter, white matter, and cerebrospinal fluid. The activated WM volume of each contact was then computed by overlapped volume between the segmented WM tissue map and the patient-specific VTA.

### Statistical analyses

Effect of target location in the SCC on stimulation evoked cortical electrophysiology. The effect of contact location within the SCC region on SCC-EP amplitude, spectral power and ITPC was assessed using a repeated measures analysis of variance (rmANOVA) with a four-level factor representing contact location along the dorsal to ventral axis of the implanted electrode and a two-level factor representing the hemisphere that received unilateral DBS. Following Waters et al. (2018) data extracted for statistical analyses was an average across frontopolar channels (18, 25, 31, 32, 37) in a time-of-interest (TOI) coincident with the reported SCC-EP feature at its negative-going amplitude maxima (50–150 ms post-pulse). Analyses were conducted in IBM SPSS 26.0.0.2 (Mathworks, Armonk, NY).

To aid in the interpretation of results, we looked at the quantity of white matter (WM) activated by each deep brain electrode contact, and tested for a relationship with the magnitude of the evoked electrophysiological response. Our hypothesis was that greater activation of conductive brain tissue (i.e., WM) would lead to a more pronounced physiological effect being recorded at the head surface. The regression analysis was conducted using the *lme4* package in R.

## Results

### Electrophysiology

Using an averaged evoked-potential approach to analysis of the cortical response to single-pulse stimulation (Figure 2A), the effect of stimulation location in the SCC (dorsal-most to ventral most contracts labeled as E3 to E0) on the magnitude of the SCC-EP feature (maximal ∼100 ms) was statistically significant, *F*(3, 18) = 4.868, *p* = 0.012, partial eta^2 = 0.448, while the effect of hemisphere (which hemisphere received SCC DBS) was not, *p* = 0.345 (Figure 2B). The interaction of hemisphere and contact factors was below threshold for statistical significance, *p* = 0.250. Results of a within-subjects contrast indicated linear model fit to changes in mean amplitude, which decreased with stimulation along the dorsal to ventral axis of implanted contact, *F*(1, 6) = 6.933, *p* = 0.039, eta = 0.536. Figure 2A shows the topography of SCC-EP maxima averaged across all conditions. Figure 2B shows grand average SCC-EP traces at each of eight contacts, with 95% confidence intervals bootstrapped from 1,000 iterations.

**FIGURE 3:**
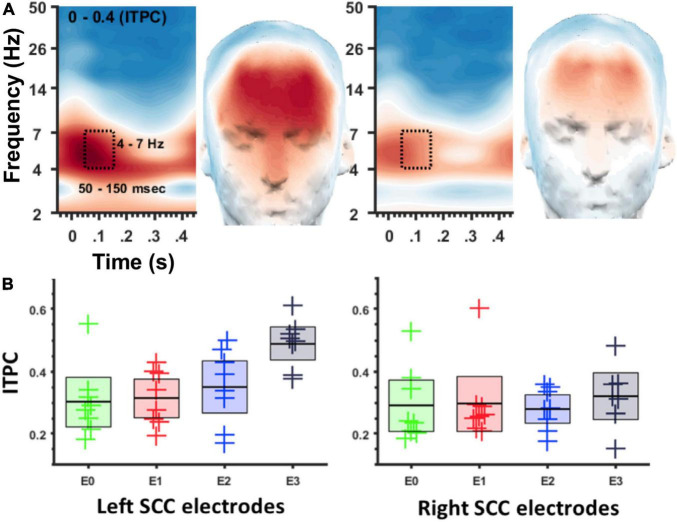
ITPC at 4–7 Hz depends on location of DBS in the SCC region. **(A)** Spectrogram of ITPC across time and frequency. Box denotes time-frequency region-of-interest used for topographic plots. Left panel for left SCC stimulation, right panel for right SCC stimulation. **(B)** Box plots showing ITPC (50–150 ms, 4–7 Hz) at different stimulation locations. Left panel for left SCC stimulation, right panel for right SCC stimulation. Green = E0/8, Red = E1/9, Blue = E2/10, Black = E3/11.

**FIGURE 4:**
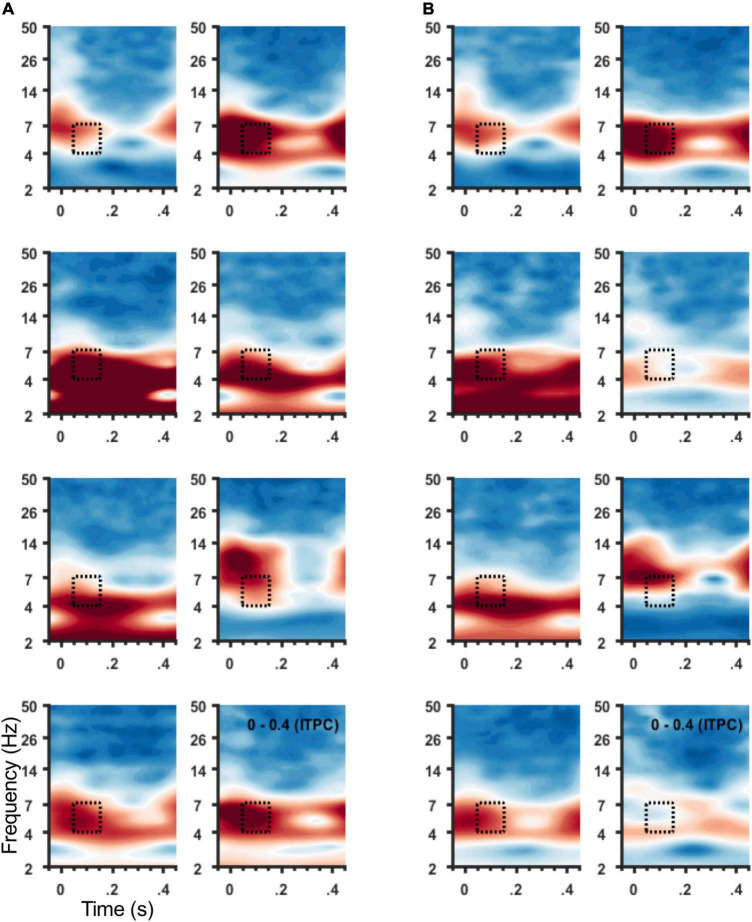
ITPC at 4–7 Hz for individual participants. Spectrograms of average ITPC across all stimulation locations from frontal sensors (Figure 1C) for individual participants. Subject order (1–8) shown within panel: right to left column then top to bottom row. **(A)** Spectrograms show ITPC time-locked to left hemisphere SCC DBS, and **(B)** to right hemisphere SCC DBS. Stippled box denotes time-frequency region of interest used for group analysis and topographic plots in **(B)**.

**FIGURE 5:**
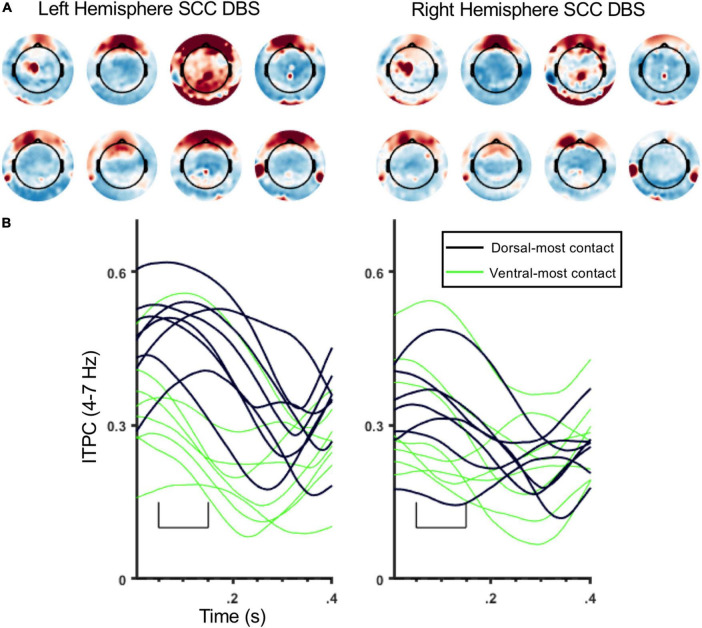
Topography and time course of ITPC at 4–7 Hz for individual participants. **(A)** ITPC topography for individual participants (4–7 Hz, 50–150 ms) averaged across all stimulation locations. The 8 topomaps on the left are from left SCC stimulation, and 8 topomaps on the right are from right SCC stimulation. Subject order (1–8) of topomaps: top rows (right to left) then bottom rows. **(B)** ITPC waveforms (4–7 Hz; frontal sensor montage, Figure 1C) from individual participants. Black lines are ITPC waveforms following stimulation at E3/11, green lines are ITPC waveforms following stimulation at E0/8. Left panel is from left SCC stimulation, and right panel is from right SCC stimulation.

ITPC in the theta band (4–7 Hz) was coincident with the SCC-EP following both right and left hemisphere stimulation (Figure 3A). The effect of stimulation location in the SCC region on the magnitude of theta ITPC was statistically significant, F(3,18) = 7.902, *p* = 0.001, partial eta^2 = 0.568, while the effect of hemisphere (which hemisphere received SCC DBS) was not, *p* = 0.103 (Figure 3B). The interaction of hemisphere and contact was significant, F(3,18) = 3.3, *p* = 0.045, eta^2 = 0.353. Mean ITPC magnitude decreased in response to stimulation along the dorsal-ventral axis of the DBS contact in the left hemisphere (E3 = 0.48, *SD* = 0.08; E2 = 0.32, *SD* = 0.11; E1 Mean = 0.30, *SD* = 0.08; E0 Mean = 0.27, *SD* = 0.05) and right hemisphere (E11 = 0.32, *SD* = 0.10; E10 = 0.28, *SD* = 0.07; E9 Mean = 0.29, *SD* = 0.13; E8 Mean = 0.25, *SD* = 0.07). Results of a within-subjects contrast indicated linear model fit to changes in mean amplitude across contacts, F(1,6) = 16.804, *p* = 0.006, eta = 0.737, with the interaction term below the significance threshold, *p* = 0.059. On the level of individual patients, theta ITPC coincident with the SCC-EP feature of the average evoked response was robust across the sample (Figure 4), including consistent spatial topography and effects of DBS location within the target region (Figure 5).

Using a time frequency approach to analysis of the cortical response to single-pulse stimulation, an increase in delta power (2–4 Hz) was observed in the study population average following both left and right stimulation but was inconsistently observed across individual subjects (Supplementary Figure 1) and thus excluded from additional analyses.

### Regression results

ITPC across all stimulation locations was significantly correlated with EP amplitude Spearman’s r(64) = -0.41, *p* < 0.001. Follow-up correlations showed that ITPC and EP were significantly correlated following left SCC perturbation r(32) = -0.57, *p* < 0.001, whereas ITPC and EP were unrelated following right SCC perturbation r(32) = -0.002.

When testing for a relationship between quantity of WM stimulated and Fpz theta ITPC, while accounting for contact position and non-independence of repeated ITPC measures in each subject, we found an association between activated WM (mm3) and ITPC, R2 = 0.13, *p* < 0.01 (Supplementary Figure 2).

## Discussion

### Findings

Using a perturbation-mapping approach, we investigated cortical time-frequency dynamics following stimulation applied to different locations of the SCC target region. Elaborating on the SCC DBS evoked potential described by Waters et al., 2018, pulse-wise perturbation was characterized by changes in delta band (2–4 Hz) power and theta band (4–7 Hz) phase alignment, coincident with the SCC-EP. Frontal theta phase alignment was observed after right or left hemisphere SCC stimulation with notable reliability; observed both at the group level and participant level. As hypothesized, millimeter scale changes in the location of stimulation also impacted cortical time-frequency dynamics: frontal theta phase clustering increased as the stimulation location was moved from ventral to dorsal contacts within the target region of the SCC, particularly when stimulation was initiated in the left hemisphere. ITPC evinced by left SCC stimulation was significantly correlated with SCC-EP magnitude. A *post hoc* correlation analysis demonstrated a trend toward a positive correlations between theta ITPC and white matter volume.

### Context/interpretation

In healthy control participants, oscillations at theta frequency (4–8 Hz) predict behavioral adaptation to errors, conflict, and novelty (Cavanagh et al., 2009; Cavanagh and Frank, 2014; Cooper et al., 2019; Duprez et al., 2020). Frontal theta oscillations are also a hypothesized mechanism of depression pathophysiology with relevance to recovery and responsivity to antidepressant medication (Arns et al., 2015; Pizzagalli et al., 2018; Whitton et al., 2019) and brain stimulation (Narushima et al., 2010; Broadway et al., 2012). Theta oscillations are pronounced across frontostriatal regions relevant to depression, especially midcingulate regions, striatum, ventral tegmental area, lateral prefrontal cortex, and hippocampus (Cavanagh et al., 2009; Cavanagh and Frank, 2014; Herweg et al., 2016; Marawar et al., 2017; Smith et al., 2020; Dede et al., 2021). The phase of theta oscillations specifically is believed to facilitate cross talk between nodes within this frontostriatal network (Cavanagh et al., 2009; Dede et al., 2021). For example, theta phase clustering is greatly enhanced across frontal regions in healthy participants after behavioral errors (Trujillo and Allen, 2007; Cavanagh and Frank, 2014), and theta phase predicts magnitude of participant’s post-error behavioral adaptation (i.e., reaction time and accuracy; Cavanagh et al., 2009; Dede et al., 2021). Brain stimulation at theta frequencies targeted at the frontal lobes has also been successfully utilized as a treatment for depression (Berlim et al., 2017), and stimulation time-locked to the phase of a participant’s frontal theta activity can enhance cognitive performance (Alagapan et al., 2019; Reinhart and Nguyen, 2019).

Enhancement of phase clustering is sometimes conceptualized as a “reset” in the timing of intrinsic brain rhythms. This phase “reset” is believed to facilitate a reorienting of attention, and/or the recruitment of brain regions important for modifying behavioral strategies (i.e., lateral prefrontal cortex; Cavanagh et al., 2009; Cavanagh and Frank, 2014). More specifically, the precise timing of a frontal theta rhythm is updated/(re)started, and this restart facilitates synchrony between brain regions demonstrating a propensity toward theta rhythm (e.g., frontostriatal regions noted above). Notably, cortical theta oscillations rely on structural pathways, and healthy participants with stronger theta tend toward larger pathway volumes across the PFC (Cohen, 2011a); conversely, reduced fractional anisotropy in individuals with head injury correlates with diminished theta-band synchrony (Cavanagh et al., 2020). Enhanced theta phase clustering can also produce ERP phenomenon (e.g., Trujillo and Allen, 2007), and present results imply ITPC contributes to the presentation of the SCC-EP. In fact, large positive correlations were observed between ITPC and EP measures in the present study, especially for left SCC stimulation. These results suggest ITPC contributes to generation of EP. This is consistent with the hypothesis that consistency in neural phase summates over experimental trials and helps generate ERPs (reviewed in Klimesch et al., 2007). Notably, prior studies examining generators of ERPs were in the context of visual/auditory stimuli or during behavioral tasks. In this regard, one speculative hypothesis is that ERPs evoked by electrical and non-electrical stimuli have overlapping biophysical (i.e., generative) mechanisms.

It has been hypothesized that electrical currents are less likely to flow through gray matter than electrically-shielded (i.e., myelin) white matter (Keller et al., 2014). Thus, it may be the case that electrical stimulation at DBS contacts near SCC gray matter (more ventral) produced a cortical response of smaller magnitude relative to DBS contacts near SCC white matter (more dorsal). A *post hoc* correlation was supportive of this possibility: ITPC amplitude showed a trend toward a positive correlation with white matter volume. Previous work has also demonstrated links between electrophysiological response magnitude and proximity of the stimulation location to white matter structures (Conner et al., 2011; Keller et al., 2014; Borich et al., 2016; Yamao et al., 2017; Nakae et al., 2019). Altogether, we are optimistic that changes in theta phase clustering represent differential activation of theta-sensitive pathways relevant for depression treatment and recovery. Future work is needed to see if features of the electrical perturbation map can further differentiate specific white matter bundles that define this therapeutic confluence point.

In the absence of acute and reliable behavioral responses to neuromodulation for psychiatric disorders, there is an urgent need for alternate methods to guide optimal parameter selection, including the position of the therapeutic contact in the target region. The SCC region is heterogeneous in terms of white matter crossing fibers (Vergani et al., 2016). Previous research demonstrated that treatment efficacy requires millimeter-scale precision of electrical stimulation at the confluence of four white matter bundles (Riva-Posse et al., 2014, 2018; Howell et al., 2019). Notably, similar approaches using stimulation pulses to guide targeting of DBS electrode placement have demonstrated promise for improved outcomes in patients with refractory conditions (Zumsteg et al., 2006; Fox et al., 2012; Entz et al., 2014; Van Gompel et al., 2015; Kimiskidis, 2016; Riva-Posse et al., 2018; Yamao et al., 2017). This line of inquiry opens new possibilities for brain mapping of structural elements in the living human brain, as well as a means to optimize and individualize the precision of brain stimulation for therapeutic purposes.

### Limitations and future directions

Despite a clear rationale, analyses in the time-frequency (TF) domain have been underutilized to observe neural oscillations in the context of perturbation mapping. This may be in part due to the challenge of disentangling stimulation artifacts from the evoked response after pre-processing. The DBS artifact is significantly greater than neurogenic activity and may covary with phase-locked EP components, obscuring modifications in neural activity that result from stimulation. Moreover, the component-based artifact mitigation used here may have attenuated some phase-locked neurogenic activity that was temporally and statistically yoked to electrical stimulation (see Smith et al., 2020 for a discussion of component-based artifact correction). This might explain discrepant findings from a simpler rejection strategy. Similarly, the absence of a relationship between ITPC and EP for right SCC stimulation is not entirely clear. Notably, Figure 2 suggests that the right SCC EP was relatively weak compared to the EP following left SCC stimulation. Research designs using symmetric biphasic pulses have the advantage of minimizing stimulator artifact on electrophysiological recordings (Liu et al., 2012) and should be considered in follow-up studies.

SCC stimulation was not randomized. This leaves open the possibility for confounding effects of stimulation location vs. stimulation sequence. This is an unfortunate consequence of experimental design, and future work will examine the influence of stimulation sequence on neural response to SCC perturbation. This confound significantly limits the conclusiveness of stimulation location effects regarding the SCC-EP. Importantly, the main findings of increased ITPC following SCC perturbation were observed regardless of SCC location.

Another important consideration in the present inquiry is the effect of current depression at time of testing: four subjects were studied after 4 weeks of treatment, and four participated after 6 months of treatment. All 8 participants were classified as responders at 6 months (HDRS depression scores reported in Supplementary Materials). A preliminary analysis suggested similar results irrespective of differences in treatment duration. Visual inspection of data from individual participants also suggests homogeneity in spatial-temporal-spectral pattern following SCC perturbation. Waters et al. (2018) also demonstrated high reliability for SCC-EP across 14 months, results arguing against a major interaction between treatment duration and response to single pulse SCC stimulation.

## Conclusion

In a small sample of SCC-DBS patients, we demonstrate the potential utility of perturbation-mapping to observe the effect of mm-level changes in DBS locations at the cortical surface. This technique has the advantage of excellent spatial and temporal resolution and holds promise as an assay of causal neural mechanisms, and may be useful for optimizing electrode placement and directing DBS current flow(s). Moreover, a time-frequency approach to analysis of single pulse electrical stimulation EP provides a view of neural phenomena that is more directly relevant to endogenous neural dynamics. Here we show theta phase coherence as a likely constituent of the SCC-EP response to SCC stimulation. Inconsistent enhancement in evoked delta power was also observed for a few participants. Evidence for stimulation evoked EEG activity as a close proxy for white matter perturbation was modest in these findings, but the approach may be promising as a read out of individual differences in cortical activity relevant to depression and treatment. These findings are generally consistent with theories of MDD etiopathogenesis that point toward frontal-lobe processes important for cognitive control, and have profound implications for the evolution of MDD treatment with neurostimulation approaches.

## Data availability statement

The data that support the findings of this study are available for research purposes upon request made to the corresponding author.

## Ethics statement

The studies involving human participants were reviewed and approved by the Emory University Department of Psychiatry and Behavioral Sciences Data Monitoring Board. The patients/participants provided their written informed consent to participate in this study.

## Author contributions

AW, ES, AV, and HM: design of inquiry. AW, AV, MO, VT, and AC: design and implementation of data collection. ES: time-frequency analyses. PR-P and AC: clinical management. KC: tractography mapping. ES, AW, BH, and AS: *post hoc* analyses. ES, AW, BH, AS, SA, CR, and HM: interpretation and preparation of the manuscript. All authors contributed to and approved the final version of the manuscript.

## Footnotes

1
http://www.fmrib.ox.ac.uk/fsl
