# Supplementary material for: Time-frequency signatures evoked by single-pulse deep brain stimulation to the subcallosal cingulate
Source: Front Hum Neurosci. 2022 Aug 18;16:939258. doi: 10.3389/fnhum.2022.939258 (PMC9433578; doi:10.3389/fnhum.2022.939258)
Supplement: Supplementary file 1 [file Data_Sheet_1.PDF]

Title: Time-frequency signatures evoked by single-pulse deep brain stimulation to the subcallosal cingulate

Authors: Ezra E. Smith, Ki Sueng Choi, Ashan Veerakumar, Mosadoluwa Obatusin, Bryan Howell, Andrew H. Smith, Vineet Tiruvadi, Andrea L. Crowell, Patricio Riva-Posse, Sankaraleengam Alagapan, Christopher Rozell, Helen S. Mayberg & Allison C. Waters

SUPPLEMENTAL MATERIALS

	Sex	Age at Surgery (years)	Study Participation (week in treatment)	Baseline HDRS-17 (mean of 4 weeks)	HDRS-17 (at time of study)
Patient 1	M	53	48	20.5	7
Patient 2	M	60	76, 139	19.25	6, 7
Patient 3	F	58	48	22.75	10
Patient 4	F	66	81	23.25	1
Patient 5	F	56	4	23.25	13
Patient 6	M	37	4	22.75	12
Patient 7	F	44	4	24.75	3
Patient 8	M	27	4	21.75	13

* Hamilton Depression Rating Scale (HDRS; Hamilton, 1960).

Supplemental Table 1. Sample Characteristics

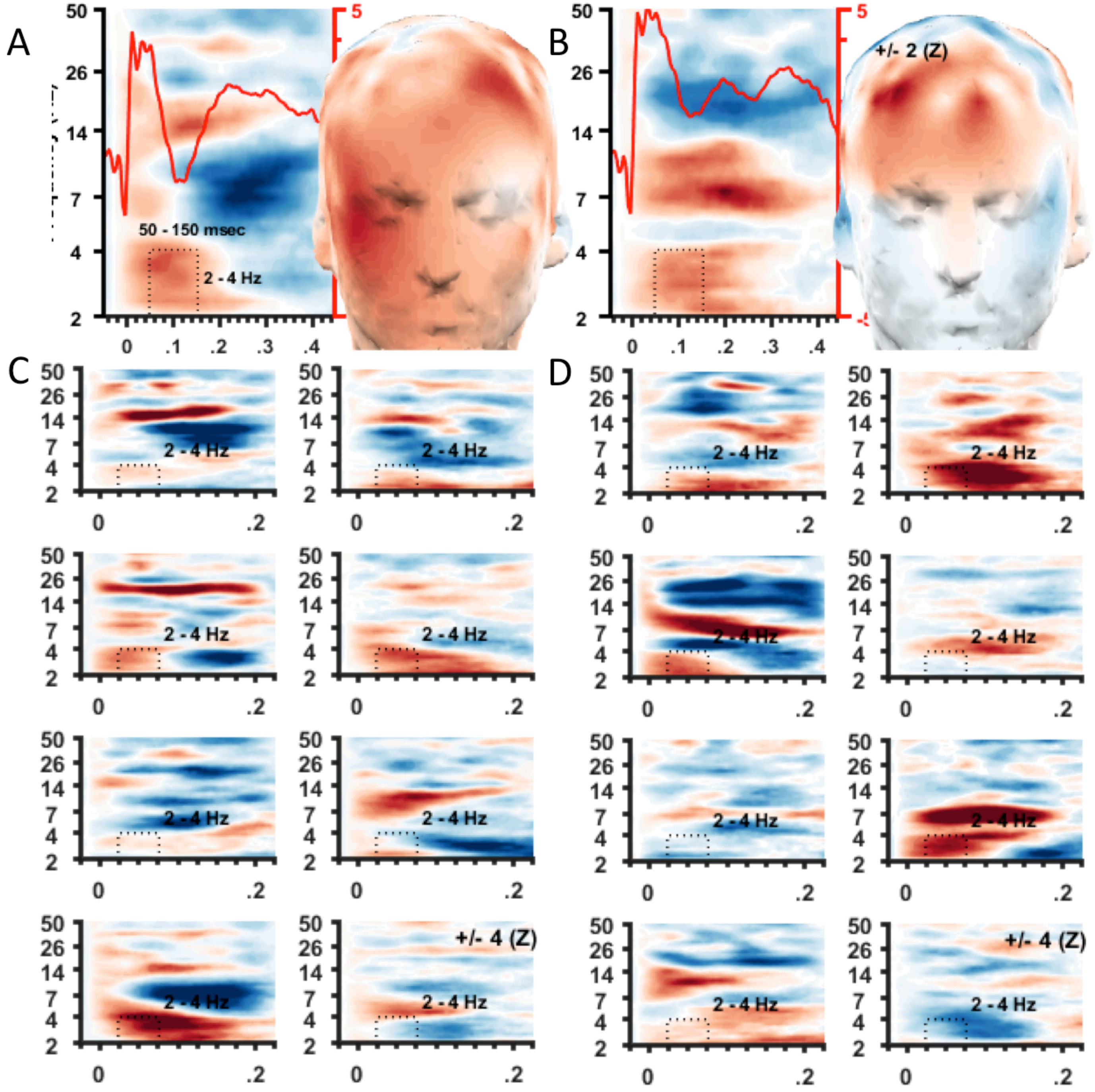

Supplemental Figure 1. Evoked delta power at 2-4 Hz was observed in the study population average following both left **(A)** and right **(B)** stimulation but was inconsistently observed across individual subjects. Spectrograms of average delta power across all stimulation locations from frontal sensors (18, 25, 31, 32, 37) for individual participants. The 8 panels on the left **(C)** are from left SCC stimulation, and 8 panels on the right **(D)** are from right SCC stimulation. Box denotes time-frequency region of interest used for group analysis and topographic plots in A,B.

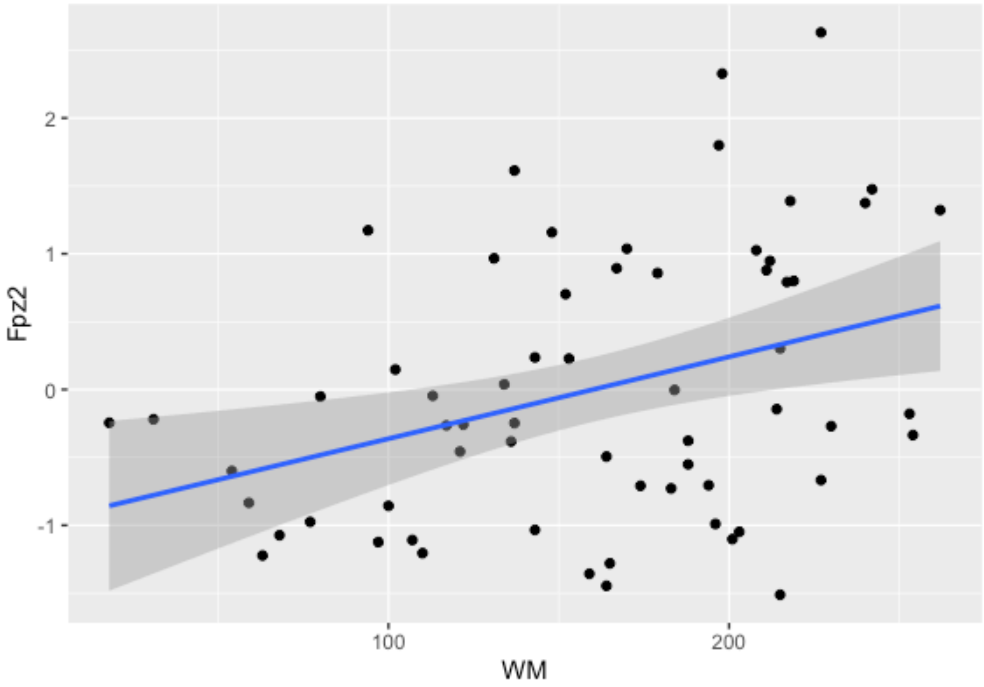

Supplemental Figure 2. When testing for a relationship between quantity of WM stimulated and Fpz theta ITPC, while accounting for contact position and non-independence of repeated ITPC measures in each subject, we found an association between activated WM (mm^3) and ITPC, R^2=.13, p<.01

Effect of Time-In-Treatment on Main effect of Perturbation Location

A confounding factor is that four subjects participated in perturbation mapping after 4 weeks of exposure to therapeutic stimulation and four participated after 24 weeks. A two-sample Student's t-test was used to compare mean theta ITPC across early and late groups (dorsal minus ventral). The null hypothesis that the group difference non-zero could not be rejected (p=.49). Given the sample size submitted to this analysis, however, this result is not definitive.

Inclusion and Exclusion Criteria for Participation in the Parent Study

All patients were enrolled at Emory University in a study of the safety and efficacy of SCC DBS for treatment resistant depression (TRD; clinicaltrials.gov #NCT01984710). Inclusion and exclusion criteria were identical to those described in Riva Posse et al., (2017), as follows: age between 18-70 years, a diagnosis of major depressive disorder, an episode of at least 12 months in length without significant response to a minimum of four adequate antidepressant treatments, lifetime failure or intolerance of electroconvulsive therapy, a 4-week average score ≥ 20 on the 17-item Hamilton Depression Rating Scale (HDRS-17; Hamilton, 1960), capacity to provide informed consent and ability to relocate to the Atlanta area for 7 months. Patients were seen for at least one month prior to surgery. After surgery, stimulation was initiated after at least four weeks, and patients were evaluated on a weekly basis for the initial six months of chronic stimulation. Standard stimulation parameters were monopolar stimulation (one contact per hemisphere), frequency=130 Hz, pulse width= 90 microseconds, and voltage starting at 3 or 3.5 V. Voltage increases were made according to the protocol if symptoms of depression were not progressing as expected.
